# Supplementary material for: Analysis of Differentially Expressed MicroRNAs in Serum and Lung Tissues from Individuals with Severe Asthma Treated with Oral Glucocorticoids
Source: Int J Mol Sci. 2023 Jan 13;24(2):1611. doi: 10.3390/ijms24021611 (PMC9864670; doi:10.3390/ijms24021611)
Supplement: Supplementary file 1 [file ijms-24-01611-s001.zip › ijms-2138665-supplementary.pdf]

Table S1. ROC curves (continuous predictors) of OCS-treated and non-OCS-treated asthmatic patients.

Predictors number	Model	AIC	AUC (95%CI)	Sens.	Spec.	H-L
1	Hsa-miR-148b-3p	4.3	0.62 (0.42, 0.82)	0.36	0.63	0.957
	**Hsa-miR-221-5p**	**8.7**	**0.75 (0.58, 0.92)**	**0.50**	**0.68**	**0.044**
	Hsa-miR-618	1.9	0.72 (0.53, 0.91)	0.36	0.74	0.503
	Hsa-miR-941	0.9	0.71 (0.53, 0.89)	0.29	0.63	0.770
	**Hsa-miR-769-5p**	**8.9**	**0.72 (0.55, 0.90)**	**0.64**	**0.63**	**0.538**
2	Hsa-miR-148b-3p + Hsa-miR-221-5p	0.6	0.72 (0.54, 0.90)	0.57	0.68	0.680
	Hsa-miR-148b-3p + Hsa-miR-618	3.4	0.64 (0.44, 0.84)	0.36	0.74	0.669
	Hsa-miR-148b-3p + Hsa-miR-941	2.2	0.68 (0.50, 0.87)	0.29	0.68	0.095
	Hsa-miR-148b-3p + Hsa-miR-769-5p	0.7	0.70 (0.52, 0.88)	0.57	0.63	0.863
	Hsa-miR-221-5p + Hsa-miR-618	9.9	0.72 (0.54, 0.90)	0.50	0.74	0.297
	Hsa-miR-221-5p + Hsa-miR-941	7.8	0.76 (0.59, 0.93)	0.43	0.63	0.935
	**Hsa-miR-221-5p + Hsa-miR-769-5p**	**7.6**	**0.77 (0.60, 0.93)**	**0.50**	**0.74**	**0.178**
	Hsa-miR-618 + Hsa-miR-941	1.9	0.68 (0.50, 0.87)	0.29	0.74	0.273
	Hsa-miR-618 + Hsa-miR-769-5p	0.5	0.70 (0.52, 0.89)	0.64	0.74	0.526
	Hsa-miR-941 + Hsa-miR-769-5p	0.2	0.70 (0.52, 0.88)	0.43	0.74	0.840
3	Hsa-miR-148b-3p + Hsa-miR-221-5p + Hsa-miR-618	1.5	0.68 (0.49, 0.87)	0.57	0.68	0.703
	Hsa-miR-148b-3p + Hsa-miR-221-5p + Hsa-miR-941	8.8	0.73 (0.55, 0.91)	0.57	0.74	0.244
	Hsa-miR-148b-3p + Hsa-miR-221-5p + Hsa-miR-769-5p	8.5	0.74 (0.57, 0.92)	0.64	0.79	0.564
	Hsa-miR-148b-3p + Hsa-miR-618 + Hsa-miR-941	3.7	0.63 (0.43, 0.82)	0.29	0.68	0.470
	Hsa-miR-148b-3p + Hsa-miR-618 + Hsa-miR-769-5p	2.4	0.67 (0.48, 0.86)	0.64	0.68	0.751
	Hsa-miR-148b-3p + Hsa-miR-941 + Hsa-miR-769-5p	2.1	0.67 (0.48, 0.85)	0.36	0.74	0.810
	Hsa-miR-221-5p + Hsa-miR-618 + Hsa-miR-941	9.8	0.71 (0.53, 0.89)	0.29	0.68	0.936
	Hsa-miR-221-5p + Hsa-miR-618 + Hsa-miR-769-5p	9.6	0.72 (0.54, 0.90)	0.50	0.68	0.171
	Hsa-miR-221-5p + Hsa-miR-941 + Hsa-miR-769-5p	9.2	0.75 (0.58, 0.92)	0.50	0.68	0.058
	Hsa-miR-618 + Hsa-miR-941 + Hsa-miR-769-5p	2.1	0.68 (0.49, 0.86)	0.43	0.74	0.552
4	Hsa-miR-148b-3p + Hsa-miR-221-5p + Hsa-miR-618 + Hsa-miR-941	0.8	0.67 (0.47, 0.86)	0.43	0.63	0.254
	Hsa-miR-148b-3p + Hsa-miR-221-5p + Hsa-miR-618 + Hsa-miR-769-5p	0.5	0.70 (0.51, 0.88)	0.64	0.79	0.562
	Hsa-miR-148b-3p + Hsa-miR-221-5p + Hsa-miR-941 + Hsa-miR-769-5p	9.8	0.73 (0.55, 0.90)	0.57	0.74	0.433
	Hsa-miR-148b-3p + Hsa-miR-618 + Hsa-miR-941 + Hsa-miR-769-5p	4.1	0.61 (0.42, 0.81)	0.36	0.74	0.771
	Hsa-miR-221-5p + Hsa-miR-618 + Hsa-miR-941 + Hsa-miR-769-5p	1.1	0.70 (0.52, 0.88)	0.36	0.68	0.948
5	Hsa-miR-148b-3p + Hsa-miR-221-5p + Hsa-miR-618 + Hsa-miR-941 + Hsa-miR-769-5p	1.8	0.68 (0.49, 0.87)	0.50	0.68	0.444

AIC: Akaike's Information Criterion; AUC: Area Under the Curve; IC: Confidence Interval; Sens.: Sensitivity; Spec.: Specificity; H-L: Hosmer-Lemeshow. Shadow, bold and underlined text correspond to the hsa-miRs included in the best multivariate logistic regression model.
